# Emotion regulation variability and flexibility in daily life show distinct associations with well-being, age, and executive functions

**DOI:** 10.1038/s41598-026-57813-7

**Published:** 2026-06-15

**Authors:** Dorian de la Fuente, Julia Karbach, Ulrike Basten, Julia A. Glombiewski, Tina In-Albon, Tanja Lischetzke, Christina Mema, Rebecca A. Rammensee, Marcel C. Schmitt, Tanja Könen

**Affiliations:** 1 Department of Psychology, RPTU University Kaiserslautern-Landau, Landau, Germany; 2 Department of Psychology, School of Social Sciences, University of Mannheim, Mannheim, Germany; 3 Mental mHealth Lab, Institute of Sport and Sports Science, Karlsruhe Institute of Technology, Karlsruhe, Germany

**Keywords:** Emotion regulation, Variability, Flexibility, Executive functions, Age, Psychology, Human behaviour

## Abstract

Flexible and effective emotion regulation (ER) is crucial for mental health and well-being. Research on individual differences in ER, however, has often relied on global self-reports or controlled laboratory settings, which may not fully capture the dynamic and context-dependent nature of regulation in daily life. To address this, we examined how ER variability and flexibility in real-world contexts relate to executive functions (EF), affective well-being, and age in a general population sample (*N* = 161, aged 14–78). EF performance was assessed using six online cognitive tasks, while ER variability and flexibility were assessed using a 14-day ambulatory assessment of daily emotional experiences. As expected, EF performance declined with increasing age across shifting, inhibition, and updating domains. Beyond these age-related differences in cognitive performance, older age was robustly associated with lower within- and between-strategy ER variability. In contrast, ER flexibility remained relatively stable across adulthood. Individual differences in EF were not associated with everyday ER variability, suggesting that cognitive control capacity does not account for regulatory dispersion in daily life. For affective well-being, ER flexibility showed specific, nonlinear associations: within-strategy flexibility based on situational valence and social context demonstrated U-shaped relations with unpleasant mood, suggesting that both low and high context sensitivity may be linked to elevated negative affect. In contrast, flexibility indices were unrelated to between-strategy variability or depressive symptoms. Overall, these findings indicate that adaptive ER cannot be reduced to greater executive control capacity, greater variability, or greater flexibility per se. Instead, they underscore the importance of distinguishing variability from flexibility and highlight that the adaptiveness of ER may depend more on the appropriateness of strategy–context matches than on general patterns of strategy use.

**Supplementary Information:**

The online version contains supplementary material available at 10.1038/s41598-026-57813-7.

## Introduction

Emotions shape our experiences, decisions, and interactions. Effective emotion regulation (ER) promotes social success, well-being, and resilience^1,2^, while difficulties in ER can increase distress and vulnerability to mental health problems such as depression, anxiety, and binge eating^3^. Specifically, rigid, context-inappropriate ER strategies are linked to issues such as depressive episodes, underscoring the need to better understand the effective use of strategies to foster mental health^4^. Traditional research classified ER strategies as either adaptive (e.g., cognitive reappraisal, acceptance) or maladaptive (e.g., suppression, rumination), based on their association with psychopathology or direct effects on emotion^5^. However, the assumption that a specific ER strategy has the same effects across different situations has been described as the *fallacy of uniform efficacy*^6^ and the effectiveness of ER strategies has been shown to depend on contextual demands^7,8^.

### Variable and flexible emotion regulation

ER flexibility refers to the ability to select and implement ER strategies in response to changing situational demands. Although ER variability and ER flexibility are often used interchangeably in the literature, there is an important conceptual distinction between the two constructs. According to Aldao et al.^9^, ER variability can be understood as a precursor to ER flexibility and describes the extent to which individuals vary their use of different ER strategies across contexts. Building on this notion, ER flexibility is determined by the joint fluctuation of variable strategy use and contextual changes, such as situational valence or social context (cf. ^8^). Importantly, flexibility has been proposed to be adaptive particularly when it supports regulatory goal attainment, for instance by facilitating the reduction of negative affect or the maintenance of well-being^10^.

At the same time, the adaptiveness of ER flexibility likely depends on its degree. Insufficient flexibility is characterized by rigid and context-insensitive regulation, reflected in a narrow and invariant use of ER strategies across situations. Research from self-reports and ambulatory assessment indicates that such rigidity is associated with poorer well-being and increased depressive symptomatology^6,11,12^. As an indirect indicator of this lack of flexibility, studies frequently refer to heightened emotional inertia (i.e., the temporal stability of affective states). Such stable and context-insensitive use of ER strategies may hinder adaptive coping in dynamic environments. Conversely, greater variability in ER does not necessarily indicate adaptiveness per se. From a goal-regulation perspective, successful regulation requires a balance between persistent goal pursuit and flexible adjustment to situational demands. Excessive shifts in regulatory goals or strategies may undermine commitment and coherence, thereby impairing effective goal attainment^13^. Correspondingly, empirical work on affective dynamics indicates that pronounced emotional instability is linked to increased psychological distress and depressive symptoms^11,14^. This suggests that emotional functioning requires a certain degree of stability. Daily-life research on emotion goal dynamics further suggests that temporal variability can reflect either context sensitivity or goal instability, depending on its extent and situational demands^15^. This trade-off between stability and flexibility is captured by the shielding–shifting dilemma, which posits that adaptive regulation requires both the maintenance of regulatory goals and the capacity to update them when circumstances change^13^. Taken together, these theoretical and empirical considerations suggest that optimal ER flexibility may lie at an intermediate level, motivating the examination of nonlinear, quadratic associations with emotional outcomes.

### EF as cognitive control mechanisms in ER

The cognitive control framework of ER proposed by Pruessner et al.^16^ builds on Gross’s extended process model of ER (2015) by specifying how executive functions (EF) may enable flexible regulation across changing contextual demands^17^. Central to this framework is the assumption that the three core EFs of shifting, inhibition, and updating constitute the primary cognitive resources underlying ER. Depending on regulatory demands, EFs are proposed to operate in a *shifting mode* that prioritizes flexibility or in a *shielding mode* that supports stable regulation^13,16^.

### Shifting

Shifting refers to the ability to flexibly switch between mental sets, tasks, or strategies^18^. In ER, shifting is engaged when ongoing regulatory efforts prove ineffective or when situational demands change, enabling disengagement from a current strategy and transition to an alternative one^16^. Accordingly, shifting is most closely associated with the shifting mode of cognitive control, which prioritizes flexibility and responsiveness to new information and supports strategy stopping and switching across dynamic emotional contexts^19^.

### Inhibition

Inhibition involves the suppression of dominant or context-inappropriate responses^20^. In ER, inhibition is particularly relevant when regulatory goals require maintaining a selected strategy despite emotional interference or competing impulses. Within the cognitive control framework, inhibition primarily supports the shielding mode of control, which emphasizes stability over flexibility and protects ongoing regulatory efforts from disruption^21^.

### Updating

Updating refers to the continuous monitoring and replacement of information in working memory^22^. In ER, updating supports both the maintenance of regulatory goals and the evaluation of regulatory outcomes. Accordingly, updating contributes to both control modes: it sustains goal-relevant information during shielding and integrates contextual feedback that signals the need for strategy change during shifting, thereby supporting monitoring processes that coordinate transitions between modes^23^.

### Integrative perspective and empirical evidence

Together, this framework conceptualizes ER as relying on three EF components that are differentially recruited to support distinct modes of control. This perspective suggests that EF—ER associations are best understood at the level of specific components, such as shifting for strategy switching or inhibition for goal shielding, rather than as a unitary construct^16,18^. Consequently, the following section reviews evidence for these selective associations and the dynamic resource trade-offs between cognitive control and regulation.

### Component specificity and resource dynamics in ER

Experimental and correlational research suggests that EF components show selective associations with ER, depending on the regulatory processes involved. For instance, Mohammed et al.^24^ demonstrated a clear dissociation in which working memory updating predicted effective cognitive reappraisal, whereas inhibitory control was selectively associated with expressive suppression. This supports the view that EF components contribute to distinct regulatory subprocesses. Similarly, Rodas and colleagues^25^ found that, among several executive measures, only shifting performance reliably predicted emotional downregulation. Recent findings^26^ confirm this selectivity, linking shifting to attentional redirection and inhibitory control to suppression. Consequently, associations often remain weak or nonsignificant when EF is treated as a unitary construct or when the EF component is misaligned with the specific regulatory demand^25,27,28^. Together, these findings challenge a simple “more EF equals better ER” account. Instead, they suggest that EF–ER associations depend on the alignment between specific executive processes and regulatory tasks.

Beyond static associations, experimental evidence points to dynamic interactions that reflect shared and limited cognitive resources between EF and ER. According to this view, regulatory efforts and executive tasks draw from the same cognitive pool, leading to performance trade-offs^29^. For example, Koay and Van Meter^30^ found that manipulating ER demands during a task-switching paradigm led to specific performance costs. While participants were actively regulating their mood, inhibitory control performance slowed significantly, suggesting that inhibitory processes were heavily recruited for the regulation task and thus less available for the concurrent executive task. These dynamic trade-offs further emphasize that the relationship between EF and ER is not just about having a certain capacity, but about how these limited resources are allocated in real time^31^. Such findings may provide a bridge to daily-life research, where the availability of executive resources may fluctuate depending on current situational demands and the intensity of the emotional experience^32^.

### Implications for the cognitive control framework

Taken together, current evidence supports a differentiated cognitive control framework of ER. Rather than contributing uniformly to regulatory outcomes, EF supports distinct regulatory processes, with shifting showing the most consistent associations. This pattern aligns with the emphasis of Pruessner et al.^16^ on adaptive control processes that enable individuals to maintain, adjust, and coordinate regulation across changing contexts. These component-specific dynamics have important implications for emotional functioning across the lifespan, during which both EF and ER undergo systematic age-related changes.

### Age, EF, and ER

Aging is accompanied by reliable declines in EF, though trajectories vary significantly across specific components. Longitudinal population-based research indicates that these changes follow complex, nonlinear trajectories; while cognitive performance often remains relatively stable through midlife, significant declines typically accelerate in later adulthood^33,34^. Importantly, longitudinal findings suggest that EF decline is selective rather than global. Contrary to the idea of a uniform reduction, updating often shows the earliest and most pronounced declines, followed by reductions in shifting and inhibitory control^35^. Furthermore, individual differences in cognitive change before old age are highly predictive of the rate of decline in later life, highlighting the importance of a lifespan perspective on cognitive resources^33^.

These component-specific EF declines have direct implications for ER implementation and monitoring. Effortful strategies such as cognitive reappraisal place substantial demands on inhibition, updating, and shifting processes, whereas less demanding strategies such as distraction rely less on executive control^17,36^. In line with these resource demands, experimental evidence shows age-related decline in the implementation of reappraisal but not distraction, as well as reduced flexibility in maintaining versus switching between strategies across emotional contexts, with these age effects being significantly mediated by EF performance^37^. Experience sampling findings further indicate that increasing age is associated with reduced variability in daily ER strategy use, while ER effectiveness and differentiation remain largely preserved^38^.

At the same time, older age is consistently associated with more positive emotional experiences^39^. Yet, age differences in ER do not seem to reflect simple shifts in regulatory preferences^40^, and evidence on age-related differences in ER strategy use remains mixed^41^. In daily life, older adults often report reduced variability in ER strategies such as cognitive reappraisal and suppression^42^, a pattern that has been interpreted as reflecting more consistent matching of strategies to situational demands rather than regulatory rigidity^43^. Within a flexibility framework, this pattern can be understood as an adaptive person–context fit: older adults may draw on a narrower but appropriate set of ER strategies because their everyday emotional contexts are more stable and better aligned with their goals. From a lifespan perspective, reduced ER variability may also reflect adaptive selection in the face of declining EF resources, indicating strategic compensation rather than diminished regulatory capacity. In line with socioemotional selectivity theory^44^, older adults also tend to proactively shape their environments by prioritizing emotionally meaningful and less conflictual contexts, which may further reduce the need for frequent strategy switching. In this sense, age-related EF declines modify the “cost structure” of flexible ER, making moment-to-moment switching less efficient and promoting forms of flexibility expressed through strategic stability and selective adjustment rather than high variability.

### The present study

The present study addresses several gaps in the literature on ER, EF, and aging. First, while age-related declines in EF are well documented, evidence on how age relates to specific EF components when examined alongside ER remains limited. We therefore examined whether increasing age is associated with poorer performance across shifting, inhibition, and updating (H1). Second, although cognitive control models suggest that EF supports variable and flexible regulation, empirical evidence linking EF to ER in daily life is scarce. We addressed this by examining component-specific associations of shifting, inhibition, and updating performance with ER variability measured using ambulatory assessment. While we specifically expected shifting to be particularly relevant for strategy transitions (H2), the associations with inhibition and updating were examined exploratively. Third, research on the link between ER flexibility and well-being has primarily focused on linear associations, despite theoretical accounts suggesting that both overly rigid and overly variable regulation may be maladaptive. Building on the trade-off between stability and flexibility, we tested whether ER flexibility shows quadratic associations with affective outcomes. Specifically, we expected ER flexibility to exhibit a U-shaped association with both average unpleasant mood (H3) and depressive symptoms (H4), such that intermediate levels of flexibility would be more favorable than very low or very high levels. Finally, we examined age-related differences in regulatory dynamics. Based on lifespan research and models of adaptive selection, we expected ER variability to decrease with age. In contrast, we expected ER flexibility to remain largely preserved, as it reflects the effective matching of strategies to situational demands (H5). All hypotheses, key variables, and operationalizations are summarized in Table 1.

**Table 1:** Summary of hypotheses and key variables.

No.	Hypothesis	Key Variables	Level/Operationalization
H1	We hypothesized that with increasing age, participants show poorer EF performance.	Age; EF (shifting, inhibition, and updating)	Person-level task performance
H2	We hypothesized that EF performance is associated with ER variability.	EF; ER variability	Person-level task performance; aggregated (person-specific) variability indices
H3	We expected ER flexibility to exhibit a U-shaped association with unpleasant mood.	ER flexibility; unpleasant mood	Person-specific flexibility slopes; aggregated mood (person-mean)
H4	We expected ER flexibility to exhibit a U-shaped association with depressive symptoms.	ER flexibility; depressive symptoms	Person-specific flexibility slopes; depressive symptoms (person-level)
H5	We hypothesized that with increasing age, participants show lower ER variability; but no association with ER flexibility was expected.	Age; ER variability; ER flexibility	Aggregated (person-specific) variability indices; person-specific flexibility slopes

Note. EF = executive functioning; ER = emotion regulation.

## Methods

To comprehensively investigate the interplay between variable and flexible ER, EF, and affective well-being across a broad age range spanning adolescence to later adulthood, we implemented a multi-method approach combining cognitive performance tasks and real-world assessments of emotional experiences. Participants completed a set of standardized online EF tasks to assess core cognitive control abilities. These were followed by a 14-day ambulatory assessment (AA) phase in which participants reported on their daily use of ER strategies and emotional states in naturalistic settings. Below, we describe the study context, procedures, measures, and analytic strategies in detail.

### Study context

This research is part of a larger project (Research Initiative Affect Regulation: Processes, Interventions, and Development in Clinical and Non-Clinical Populations, *ARPID*), investigating emotional processes in individuals with and without chronic pain, aiming to better understand cognitive and emotional mechanisms in these populations. The study was conceptualized in accordance with ethical guidelines and received approval from the local ethics committee (application # LEK-397). For the present study, we focused on individuals without chronic pain drawn from the general population to test our preregistered hypotheses (10.17605/OSF.IO/3ENQK). This approach allows for an less confounded examination of these processes, as chronic pain impacts both ER (Koechlin et al., 2018) and cognitive performance (Rathbone et al., 2016), introducing potential confounding effects such as cognitive load.

### Study procedure

After providing written informed consent (and parental consent for minors under 16), participants first completed an online trait questionnaire at home to assess their sociodemographic characteristics and trait measures. EFs were then assessed in two online sessions conducted on participants’ home computers on separate days. These two EF sessions were scheduled no more than three days apart. Following completion of the EF assessment, participants entered a 14-day AA phase. The AA phase always started on a Monday; however, there was no fixed interval between the EF assessment and the start of the AA. Instead, participants were contacted by telephone, received a standardized briefing, and scheduled the two-week AA period in coordination with the study team to ensure that the assessment could be reliably completed from the participants’s perspective. This procedure was chosen to maximize compliance and data quality while maintaining a consistent weekday-based assessment structure. Additional laboratory-based assessments were conducted as part of the broader project but are not included in the present paper. Data collection took place between April 2022 and March 2024. Participants received financial compensation of up to €150, depending on the number of completed study components.

### Executive function assessment

The EF assessment was completed in two sessions, each lasting approximately 40 min, conducted on participants’ home computers on two separate days. The tasks were designed to target three core EF domains: shifting, inhibition, and updating. In the first session, participants completed the Wisconsin Card Sorting Test (WCST^45^ to assess shifting ability, the Antisaccade Task (AST^46^ to evaluate inhibition, and the Visual-Verbal Complex Span Task (VCST^47^ to measure updating. The second session comprised the Spatial n-Back Task (SNT^48^ for updating, the Sustained Attention-to-Cue Task (SACT^49^ for inhibition, and the Flexible Item Selection Task (FIST^50^ for shifting.

### AA phase

Following the EF assessment, participants began the 14-day AA phase. We followed the reporting guidelines for AA studies outlined by Trull and Ebner-Priemer^51^. For this phase, participants installed one of two mobile applications—SEMA3 (O’Brien et al., 2024) or m-Path^52^—depending on their smartphone operating system. They selected one of two daily schedules (8:00 a.m. to 9:00 p.m. or 9:00 a.m. to 10:00 p.m. on weekdays, with a one-hour later start time on weekends). A stratified random interval scheme was employed, prompting participants five times daily to complete brief surveys lasting approximately five minutes each. Notifications remained active for 30 min, with a reminder issued after 15 min if unanswered.

### Participants

Participants were recruited throughout Germany using electronic and print advertisements as well as social media and completed all assessments in German. Interested individuals registered on the study website, where detailed information about the study was provided. Inclusion criteria were being at least 14 years old (parental consent was required for individuals under 16), having access to a computer with a keyboard and mouse to complete online cognitive tasks, and owning a smartphone for the AA phase. The final sample consisted of 161 participants (44.1% male, 55.3% female, and 0.6% diverse), with a mean age of 39.6 years (SD = 15.9, range = 14–78 years). To ensure a broad representation of different age groups, participants were recruited across various age ranges: 14–19 years (10.6%, *n* = 17), 20–29 years (19.3%, *n* = 31), 30–39 years (24.2%, *n* = 39), 40–49 years (19.3%, *n* = 31), 50–59 years (12.4%, *n* = 20), 60–69 years (10.6%, *n* = 17), and 70–79 years (3.7%, *n* = 6). For all analyses, however, age was treated as a continuous variable. Although the sample covered a broad age range, the number of participants in later adulthood was comparatively small. Regarding education, the majority of participants had completed a higher secondary school diploma (32.3%), a master’s or equivalent degree (29.8%), or a bachelor’s degree (20.5%). A smaller proportion reported completing lower secondary school (11.8%), while 3.7% had obtained a doctoral degree. 1.2% of participants reported completing lower secondary school after 8–9 years, and 0.6% did not provide information on their educational attainment. With respect to occupational status, the largest proportion of participants identified as employees (43.5%), followed by students (19.3%) and retirees or pensioners (11.8%). Smaller proportions reported being civil servants (8.1%), self-employed (3.7%), or engaged in other occupations (3.7%). Additionally, 5.6% were school students, whereas 1.2% each reported being apprentices, unemployed, or homemakers. One participant (0.6%) did not report their occupational status.

### Data cleaning

To preprocess the EF data, we applied cleaning procedures at both the trial and task levels prior to calculating task scores. Trials with response times shorter than 200 ms, indicative of potential careless responding, were excluded. Additionally, trials exceeding 2.5 standard deviations above or below a participant’s mean response time for a given task were removed as outliers. Task scores below chance performance thresholds were also excluded to ensure meaningful participant engagement with the tasks. EF task data were available for the following numbers of participants: WCST (*n *= 152), AST (*n *= 148), VCST (*n *= 155), SNT (*n *= 157), SACT (*n *= 154), and FIST (*n *= 158).

Of the 176 individuals who initially registered for the AA phase, five were excluded due to erroneous assignment, leaving 171 participants. An additional three participants were removed for not initiating any surveys, resulting in a sample of 168 participants who provided a total of 8,685 AA prompts. Each assessment took approximately 4–5 min to complete (*M* = 4.5 min). To enhance data quality, several data cleaning steps were applied at the assessment level, including the exclusion of assessments recorded too close to a previous prompt (indicating technical errors), assessments with extremely short response times, or inconsistent mood scale responses (both indicating careless responding). Because ambulatory data were aggregated at the daily level, adherence was defined at the day level, consistent with recommendations for intensive longitudinal designs^51^. Conducting multiple assessments per day ensures that day-to-day variability can be meaningfully captured, and requiring multiple valid days supports stable person-level estimates^53,54^. Notably, 62.9% of participants provided valid data for all 14 study days (*M* = 12.22, *SD* = 3.28).

### Sample size considerations

The present study was part of a larger project with multiple research questions, targeting a minimum sample size of 120 individuals without chronic pain (ARPID, see above). The final sample consisted of 161 individuals, reflecting the outcome of the overarching recruitment process. Power analyses using the R package WebPower^55^ confirmed that the final sample size (*N* = 161) provided sufficient power (> 0.80) to detect small-to-medium effects (f² = 0.09) in a multiple regression model with six predictors at a significance level of *α* = 0.05. This power estimate refers to the detection of effects based on the deviation of the overall *R²* from zero, ensuring that meaningful overall model effects can be identified. Additionally, for bivariate correlations, the sample provided power > 0.80 to detect correlations of *r* = .21 or larger (*α* = 0.05, two-tailed).

## Measures

### Online trait questionnaire

#### Depressive symptoms

Depressive symptoms were assessed using the nine-item self-report depression module of the Patient Health Questionnaire (PHQ-9^56^; German version by^57^. Participants rated the frequency of nine depressive symptoms during the past two weeks on a 4-point Likert scale ranging from 0 ("not at all") to 3 ("almost every day"). We calculated sum scores across all items as there were no missing items for any participant. Total scores of 5, 10, 15, and 20 represent cut-off points for mild, moderate, moderately severe, and severe depression, respectively^58^. Cronbach’s alpha for this sample was α = .76.

#### Executive functions

For each EF task, accuracy scores were calculated as the percentage of correct responses, rather than using measures of response time differences, to increase reliability^49,59^. The three EF domains—shifting, inhibition, and updating—were each assessed using two tasks. Domain scores were computed as the mean of the accuracy scores from the respective pair of tasks for each domain. We additionally computed an overall EF score (EF_overall_), combining all six tasks (Cronbach’s α = .73). Descriptive statistics and estimates of split-half reliability are provided in Table 2. No participants were excluded from any EF task based on practice performance, and all participants proceeded to the experimental phases.

**Table 2:** Descriptive statistics for the EF tasks, domain factors, and overall EF performance.

EF Task	*N*	M	SD	Min-Max observed	Skewness	Kurtosis	Split-Half Reliability
Shifting	150	70.53	13.66	10.42–91.95	−1.32	2.25	-
WCST	152	81.5	7.1	32.8–89.4	−4.01	22.7	0.95
FIST	158	61.1	21.8	2.08–97.9	−0.70	−0.22	0.74
Inhibition	146	82.22	12.21	34.72–98.61	−1.37	2.22	-
AST	148	80.0	14.0	36.1–98.6	−1.06	0.43	0.96
SACT	154	84.8	16.0	25–100	−2.14	5.03	0.95
Updating	153	84.09	7.69	51.95–97.56	−1.35	2.45	-
VCST	155	83.0	10.4	41.1–97.1	−1.71	3.26	0.83
SNT	157	85.6	7.7	52.0–100	−1.23	2.89	0.90
EF_overall_	161	78.7	9.66	35.2–92.3	−1.35	2.83	-

Note. Performances in the EF tasks are expressed as the percentage of correctly completed trials. Scores were averaged at the domain level (Shifting, Inhibition, Updating) and also aggregated into a composite score (EF_overall_). Estimates of split-half reliability are Spearman-Brown corrected.

### Shifting

#### Wisconsin Card Sorting Test (WCST)

In the WCST^45^, participants were presented with a card displaying one to four colored symbols (red, green, yellow, or blue; triangle, star, cross, or circle). Their task was to match each card to one of four possible piles displayed at the top of the screen. The matching criterion (e.g., by color or by the number of symbols) was not disclosed and had to be inferred through feedback provided after each trial. Once participants correctly identified and applied the matching criterion for 10 consecutive trials, the criterion changed without any indication. Participants were then required to use feedback to discover the new criterion. This process continued across multiple rule sets. The task began with a practice phase involving up to 30 trials and two rule sets to familiarize participants with the procedure. In the test phase, participants completed up to 128 trials encompassing six rule sets. The order of the target piles was consistent across all participants. The practice phase was not performance-contingent: participants completed a number of practice trials (22–30 trials across up to two rule blocks) and proceeded to the test phase regardless of accuracy or whether they successfully identified the sorting rule.

#### Flexible Item Selection Task (FIST)

In the FIST^50^, participants were presented with four cards, each displaying a combination of the attribute’s motif (ship, rabbit, or rose), size (large, medium, or small), quantity (1, 2, or 3), and color (green, red, or blue). Within each trial, participants had 14 s to form four pairs of cards, ensuring that the matching category (e.g., quantity, color, motif, or size) changed with each pairing. After selecting each pair, participants were required to identify the matching category. The task consisted of 12 test trials. Responses were made via mouse clicks on the cards and corresponding category buttons. The 14-second time limit applied to the entire trial, including the selection of matching categories. The position of the category buttons changed randomly after each pairing. The task did not include a practice phase; participants received detailed illustrated instructions before beginning the experimental trials.

### Inhibition

#### Antisaccade Task (AST)

In this task^46^, participants were instructed to accurately identify visual target stimuli while performing controlled eye movements. After fixating on a central fixation cross, a positional cue (“=”) appeared either to the left or right of the cross. Shortly thereafter (300 ms), a target stimulus (“B,” “P,” or “R”) was briefly presented (150 ms) on the opposite side of the cue (e.g., to the right of the cross if the cue appeared on the left). Participants were required to shift their gaze to the opposite side of the cue upon its appearance to detect and correctly identify the target letter. The task began with 18 response mapping trials, during which both the cue and target were centrally presented. Each target letter was presented six times, with varying fixation timings (200, 600, 1000, 1400, 1800, and 2200 ms, each presented three times). This was followed by 18 practice trials, with 50% of the targets appearing on the left. Each target letter was presented six times, with fixation timings of 200, 1000, and 2200 ms (each presented six times). Participants proceeded to the test phase regardless of practice accuracy. The main task consisted of 72 antisaccade trials, divided into three blocks of 24 trials each, with self-determined breaks between blocks. Responses were recorded using the numeric keypad, with participants pressing “7” for “B,” “8” for “P,” and “9” for “R”.

#### Sustained Attention-To-Cue Task

In the SACT^49^, participants were required to maintain their attention on a specific screen location indicated by a bright circle that gradually shrank. The circle served as a positional cue for a subsequent 3 × 3 letter matrix, from which participants had to identify the central letter. However, before the matrix appeared, a centrally presented distractor (“*”) was displayed. The matrix appeared 300 ms after the distractor at the location previously indicated by the circle. Participants were instructed to keep their attention focused on the circle’s position and avoid being distracted, enabling them to accurately identify the central letter in the matrix. The task began with a practice phase consisting of four trials using cue durations of 2000, 4000, 8000, and 12,000 ms, with feedback provided after each trial, and did not include accuracy thresholds. This was followed by a test phase with 64 trials, divided into two blocks of 32 trials each, with self-determined breaks between blocks. Cue durations (2000, 4000, 8000, and 12,000 ms) were evenly distributed across trials, and no feedback was given during the test phase.

### Updating

#### Visual Complex Span Task (VCST)

Participants were required to simultaneously perform a categorization task and maintain the sequential order of stimuli in memory^47^. Animal images were presented sequentially, either upright or upside-down. Participants indicated the orientation of each image by clicking the left or right mouse button, while also memorizing the order of the images. After the sequence was completed, all the animal images were displayed in the upper half of the screen, and participants reconstructed the sequence by clicking on the images in the correct order. The task began with a practice phase consisting of six trials at memory loads of 2, 3, and 4 items (two trials per load). After each trial, participants received feedback on their performance, including immediate feedback after recall and error feedback for incorrect categorizations during encoding. At the end of the practice phase, participants were provided with summary feedback, including their total score and a high-score load. If recall errors exceeded 50%, participants completed an additional practice block with six more trials. After this single repetition, all participants proceeded to the test phase regardless of performance. The test phase consisted of 18 trials, divided into two blocks of nine trials each, with self-determined breaks between blocks. In the first block, memory loads of 2, 3, and 4 items were presented (three trials per load). In the second block, the memory loads increased to 5, 6, and 7 items (three trials per load). Summary feedback was provided at the end of each block to inform participants of their overall performance. The percentage score included correct responses from both the encoding and recall phases.

#### Spatial *N*-Back Task

In the SNT^48^, participants were required to monitor and remember the positions of stimuli presented sequentially in a 4 × 4 grid. The stimuli, represented as flowers, appeared at various locations, and participants were instructed to press a response button whenever a stimulus reappeared in the same position as it had *n* trials earlier. The task began with a practice phase consisting of two blocks, one for the 2-back and one for the 3-back condition, and did not include performance-based thresholds or repetition. This was followed by a test phase comprising four blocks, with two blocks each for the 2-back and 3-back conditions. The stimuli were presented at random positions within the grid, with specific constraints to ensure systematic variation. Each block included 12 target items, defined as stimuli appearing in the same position as an item *n* trials earlier. Additionally, three stimuli in each block served as lures—stimuli that would be correct in position *n*, but occur at positions *n-1* or *n* + 1 in the sequence. No lure exceeded a delay of six trials, and flowers never appeared consecutively in the same position. Participants’ responses were measured as hits (correctly identified targets), omissions (missed targets), correct rejections (accurate non-target identification), and false alarms (incorrect non-target responses). The score for the SNT was calculated as the difference between correct responses (hits and correct rejections) and incorrect responses (false alarms and omission), expressed as a percentage of all trials.

### Ambulatory assessment

#### ER strategies

The use of ER strategies was assessed using ten items from Grommisch and colleagues^62^ and one additional item, avoidance, from the Heidelberg Form for Emotion Regulation Strategies^60^. The following eight ER strategies were included to capture all stages of Gross’s process model of ER^61^: *situation selection* (“I chose which situation to put myself in”), *situation modification* (“I actively changed something in the situation”), *distraction* (“I did something to distract myself (physically or mentally)”), *reappraisal-change* (“I changed the way I was thinking about the situation”), *reappraisal-perspective* (“I took a step back and looked at things from a different perspective”), *rumination* (“I thought over and over again about my emotions”), *social sharing* (“I talked with someone about my emotions”), and *suppression* (“I was careful not to express my emotions to others”). Additionally, *acceptance* (“I accepted my emotions as valid and important”) and the additional item *avoidance* (“I made sure to avoid situations that could evoke unpleasant emotions”) were assessed, along with the strategy *ignoring *(“I ignored my emotions”). Compared to Grommisch et al.^62^, two modifications were made: participants rated their strategy use over the past two hours instead of “since the last survey,” and the response format was changed from the original scale (0 = “not at all”; 100 = “very much”) to a slider ranging from 0 = “does not apply at all” to 100 = “applies very well”.

#### Context variables

At each AA prompt, participants were instructed to respond with reference to their situation or environment during the preceding two hours. All situation-related items (e.g., valence and social context) referred to this two-hour time window.

#### Valence of the situation

At each measurement occasion, participants rated the valence of the situation on a slider (“How positive or negative was what happened in the last 2 h?"), ranging from 0 (negative) to 100 (positive).

#### Social context

To determine the type of social context, participants responded at each prompt to the item, “Who have you spent the most time with in the last 2 hours?” by selecting from nine options: 0 = “alone”, 1 = “friends”, 2 = “partner”, 3 = “family”, 4 = “colleagues”, 5 = “acquaintances”, 6 = “roommates”, 7 = “strangers”, or 8 = “other”. From these responses, two complementary binary indicators were created at the prompt level: one representing being alone (0/1) and one representing being with others (0/1). For analyses at the daily level, these binary indicators were aggregated by computing the proportion of prompts within a given day in which participants reported being alone or being with others. These daily proportion scores were person-mean centered and thus reflect deviations from each participant’s typical level of being alone or being with others across the assessment period. The centered daily proportions served as continuous context predictors in the main analyses. On average, participants reported being alone during 1.31 instances per day and being with others during 2.53 instances per day, representing 34.8% and 65.2% of their total daily reports, respectively.

### Momentary mood

#### Pleasant–unpleasant mood

Momentary pleasant-unpleasant mood was assessed with an adapted short version of the Multidimensional Mood Questionnaire (MDBF^63^). Following the instruction “At this moment I feel…”, participants responded to two items (“unwell–well” [reverse-scored] and “good–bad”) using bipolar slider scales (0–100). The items were averaged, with higher scores indicating a greater degree of unpleasant mood. Given the nested data structure and the two-item scale, reliability was estimated separately at the within- and between-person level based on level-specific inter-item correlations (cf. ^64^). Reliability was high (between-person *α* = .98; within-person *α* = .86).

#### Calm–tense mood

Momentary calm–tense mood (i.e., tense arousal) was assessed with an adapted short version of the MDBF^63^, which has been used in previous AA studies (e.g., ^65^). Using the same instruction “At this moment I feel…”, participants responded to the items “relaxed–tense” and “restless–calm” (reverse-scored) on sliders from 0 to 100. Higher scores indicated greater tense arousal. Reliability was likewise high (between-person α = .96; within-person α = .81). Following Paul and colleagues^8^, this measure was included as a covariate to control for confounding effects of arousal on ER strategy use^66^.

#### Calculation of ER variability

To capture variability in the use of ER strategies, we calculated two indices: within-strategy variability and between-strategy variability (cf. ^9,^^67^). Both indices were computed at the daily level by aggregating up to five measurement occasions per day, resulting in a multilevel structure of 14 days (Level 1) nested within participants (Level 2). This approach reduces momentary fluctuations and measurement noise while preserving meaningful variability at the daily and person levels.

Within-strategy variability reflects the extent to which the use of a specific ER strategy fluctuates over time. It was calculated as the day-level standard deviation (SD) of each strategy’s use across all daily measurement occasions. These daily SDs were then averaged across all strategies and across the 14-day assessment period for each participant, yielding person-level within-strategy variability scores. Higher values indicate that the same strategy was used with substantially varying intensity across prompts within a day, whereas lower values indicate that a strategy was applied with relatively stable intensity across time. A value of zero indicates that a given strategy was reported with identical intensity across all prompts within a day, reflecting perfectly stable use.

Between-strategy variability reflects the extent to which participants prioritized strategies at a given moment. It was calculated by first determining the SD of ratings across all 11 assessed ER strategies at each measurement occasion, capturing the extent to which certain strategies were emphasized over others. These moment-level SDs were averaged within each day and subsequently across the 14 days for each participant to obtain person-level between-strategy variability scores. Higher between-strategy variability scores reflect a more selective strategy profile in which one or two strategies were emphasized much more strongly than others at a given moment, whereas lower between-strategy variability scores indicate a more balanced distribution of strategy use across multiple strategies. A value of zero indicates that all strategies received identical ratings at a given prompt. Illustrative examples are shown in Supplementary Figure S1.

Internal consistencies, as indicated by Cronbach’s alpha, were high for both indices: within-strategy variability (α = .97) and between-strategy variability (α = .97). Descriptive statistics showed that the average within-strategy variability was *M* = 25.24 (*SD* = 9.46), whereas between-strategy variability had an average of *M* = 25.74 (*SD* = 6.15). Intraclass correlation coefficients (ICCs) indicated that 74.9% of the variance in within-strategy variability and 72.5% of the variance in between-strategy variability were attributable to between-person differences, with the remaining variance arising from within-person fluctuations and measurement error.

#### Calculation of ER flexibility

ER flexibility was operationalized through two-level models (days at Level 1 nested within individuals at Level 2), constructed using the lme4 package^68^, employing maximum likelihood estimation and the bobyqa optimizer (cf. ^8^). Two separate models were estimated: one for within-strategy variability and one for between-strategy variability. These models examined the within-person associations between contextual factors (Level-1 predictors: valence, being alone, and being with others; all entered simultaneously) and ER variability (Level-1 outcomes).

Prior to analysis, all Level-1 predictors and outcomes were person-mean centered to ensure that the estimates reflect “pure” within-person associations, unaffected by stable between-person differences. To capture individual differences in contextual responsiveness, random slopes for the context variables were included. We extracted the Level-2 residuals of the random slopes (slope residual scores) as our primary indices of ER flexibility, yielding six indices of ER flexibility per participant. Although these models were estimated using within-person variation, they were applied to derive between-person estimates rather than to model intraindividual regulatory processes. The extracted Level-2 residuals of slopes were subsequently treated as between-person characteristics in all further analyses^69–71^. These scores represent the individual deviations from the average sample effect; a positive slope residual indicates that an individual’s ER strategy variability increased more in response to contextual changes compared to the average, suggesting greater flexibility. To ensure robustness of these indices, the models included day of study (to address potential reactivity effects), person-mean-centered tense arousal, and mean ER strategy endorsement (person-mean-centered) as additional Level-1 predictors. Including daily strategy endorsement accounts for the dependency between strategy use intensity and opportunity for variability (cf.^67^). This parsimonious modeling approach was chosen to ensure model stability and reliable estimation of the random effects. The extracted slope residuals were subsequently used to test for quadratic (U-shaped) associations with unpleasant mood and depressive symptoms.

#### Analytic strategy

All analyses were conducted using R software^72^. Hypotheses were tested at the between-person level using person-level indices of EF, ER variability, ER flexibility, and affective well-being. Throughout all analyses, the terms “predictor” and “outcome variable” are used for analytical clarity and do not imply causal relationships.

To test Hypothesis 1, Spearman’s rank-order correlations (*ρ*) were used to examine bivariate associations between age and EF (shifting, inhibition, updating, and the composite EF score). Spearman’s *ρ* was chosen due to its robustness to outliers and its suitability for detecting monotonic relationships without assuming normality or linearity^73^. For Hypotheses 2 through 5, multiple linear regression models were employed at the person level. Continuous between-person variables (e.g., depressive symptoms and unpleasant mood) were grand-mean centered. For Hypothesis 2, within- and between-strategy variability were regressed on the EF components, which were entered simultaneously to account for shared variance. These models additionally controlled for age, depressive symptoms, and mean ER strategy endorsement. For Hypotheses 3 and 4, regression models examined associations between ER flexibility and affective well-being (unpleasant mood and depressive symptoms). The flexibility indices derived from the multilevel models were entered as predictors, including both linear and quadratic terms to test the hypothesized U-shaped relationships. Separate models were estimated for flexibility indices based on valence and social contexts (being alone and being with others). Finally, for Hypothesis 5, linear regression models were used to examine age-related differences in ER variability and ER flexibility. Models predicting ER variability included EF performance and mean ER strategy endorsement as covariates. In models predicting ER flexibility, EF was included as a covariate, whereas mean ER strategy endorsement was not, as its influence had already been accounted for during the estimation of the flexibility slopes (see *Calculation of ER flexibility*).

#### Transparency and openness

We reported how we determined our sample size, all data exclusions, and all measures in the study. The design, hypotheses, and analyses of the present study were preregistered before conducting any statistical analyses (10.17605/OSF.IO/3ENQK). Adjustments were made to the operationalization of ER flexibility due to challenges with model convergence in the original approach. Initially, we aimed to calculate flexibility scores based on the framework proposed by Aldao et al.^9^, regressing ER variability on changes in the environment in a two-level multilevel model and saving residuals for each participant as individual flexibility scores. However, this method led to convergence issues. To address this, we adapted the approach by extracting person-specific slopes from multilevel models (as described in the Methods section). Unlike residuals, which measure deviations from expected values, these slopes provide a direct measure of the strength of association between ER variability and contextual changes, serving as interpretable flexibility scores. This approach allowed us to assess how participants adapted their ER strategies over time in response to varying contexts. Additionally, we reduced the categories of social context (from *alone*, *close*, and *non-close others* to a binary distinction between *being alone* and *being with others*) due to difficulties in differentiating close vs. non-close others (e.g., “colleagues” and “roommates” could fall into either category).

## Results

We report descriptive statistics for all key variables, followed by correlation and regression analyses testing our preregistered hypotheses on the relationships between EF, ER, affective well-being, and age. All analyses were conducted at the between-person level unless noted otherwise, and results include unstandardized and standardized coefficients, significance levels, and confidence intervals. Analyses addressing the preregistered hypotheses were theory-driven and involved a limited number of planned tests; therefore, no additional correction for multiple comparisons was applied. For exploratory analyses, including the correlation matrix, *p*-values were adjusted using the Benjamini–Hochberg false discovery rate procedure.

### Descriptive analyses

Descriptive statistics and reliabilities for the EF tasks are summarized in Table 2, while those for ER variability, context factors, and well-being measures are presented in Table 3. Bivariate correlations among key study variables are reported in Table 4, with the extended correlation matrix available in Supplementary Table S1. Depressive symptoms were primarily concentrated in the minimal to mild range (*M* = 4.64, *SD* = 3.31; see Supplementary Figure S2). No issues of multicollinearity were detected in the regression analyses.

**Table 3:** Descriptive statistics (means, SDs, cronbach’s alpha) for emotion regulation variability, context factors, and well-being.

Variables	M	Min - Max	SD_overall_	SD_between_	SD_within_	α_between_
ER variability						
Within	25.24	0–100	9.46	8.31	4.57	0.97
Between	25.74	0–100	6.15	5.33	3.08	0.97
Context						
Valence	70.22	0–100	16.42	11.88	11.12	0.84
Being alone	1.31	0–5	1.34	0.87	0.98	0.76
Being with others	2.56	0–5	1.48	1.04	1.07	0.78
Unpleasant mood	25.90	0–100	17.07	13.07	10.15	0.95
PHQ	4.64	0–27	3.31	-	-	0.76

Note. For Between-Strategy Variability, *SD*_between_ and *SD*_within_ were calculated based on the occasion-level values. For Within-Strategy Variability, Valence, Being alone, Being with others and unpleasant mood, *SD*_between_ and *SD*_within_ were calculated based on daily averages. Reliability estimates reflect two-level (within- and between-person) reliability coefficients following multilevel reliability procedures. Values reported in the table represent between-person reliability. *n* = 8,359 assessments at Level 1 and *N* = 161 participants at Level 2.

**Table 4:** Correlations among key study variables.

Variable	1	2	3	4	5	6	7	8	9
1. Age	—								
2. Depressive symptoms (PHQ-9)	− 0.35**	—							
3. EF performance (overall)	− 0.47**	0.04	—						
4. Within-strategy variability	− 0.06	0.06	− 0.06	—					
5. Between-strategy variability	0.06	0.02	− 0.12	0.91**	—				
6. Mean ER strategy endorsement	0.25**	− 0.05	− 0.21*	0.76**	0.91**	—			
7. Unpleasant mood (daily mean)	− 0.11	0.44**	− 0.09	0.07	0.00	− 0.08	—		
8. Flexibility (within-strategy; valence)	0.02	− 0.03	− 0.07	− 0.47**	− 0.43**	− 0.34**	0.07	—	
9. Flexibility (within-strategy; with others)	− 0.07	0.07	− 0.01	0.87**	0.84**	0.70**	− 0.00	− 0.78**	—

*Note. N* = 161. Spearman rank-order correlations are reported. *p* <.05*, *p* <.01**. *p*-values were adjusted using the Benjamini–Hochberg false discovery rate procedure.

### H1: correlations between EF and age

Spearman’s rank-order correlations revealed significant negative associations between age and all EF measures. Higher age was associated with lower performance in shifting (*ρ* = −0.51, *p* <.001), inhibition (*ρ* = −0.25, *p* =.002), and updating (*ρ* = −0.43, *p* <.001). In addition, age was negatively correlated with overall EF performance (*ρ* = −0.47, *p* <.001). Corresponding scatterplots with nonparametric smoothers are shown in Supplementary Figure S3. These findings are in line with our hypothesis that higher age is associated with poorer EFs, both at the level of the individual EF domains and overall EF performance.

### H2: associations of EF and ER variability

We hypothesized that shifting performance would be positively associated with ER variability. In addition, we exploratively examined whether inhibition and updating performance also relate to ER variability. To test this, we conducted multiple regression analyses predicting ER variability from EF performance while controlling for age, depressive symptoms, and mean strategy endorsement. Contrary to our expectations, EF performance was not significantly associated with ER variability when accounting for covariates. Specifically, shifting (*β* = −0.04, *p* =.53), inhibition (*β* = −0.03, *p* =.58), and updating (*β* = 0.01, *p* =.86) did not predict within-strategy variability. Similarly, neither shifting (*β* = −0.07, *p* =.18), inhibition (*β* = 0.02, *p* =.71), nor updating (*β* = 0.06, *p* =.26) emerged as significant predictors of between-strategy variability. Likewise, the overall EF composite score was not significantly associated with either within-strategy variability (*β* = −0.02, *p* =.77) or between-strategy variability (*β* = 0.01, *p* =.84). Across all models, age showed a consistent negative association with both within-strategy (*β* = −0.38, *p* <.001) and between-strategy variability (*β* = −0.31, *p* <.001). Mean ER strategy endorsement was strongly and positively associated with both within-strategy (*β* = 0.84, *p* <.001) and between-strategy variability (*β* = 0.93, *p* <.001). Finally, higher depressive symptoms were associated with lower within-strategy variability in the facet-level model (*β* = −0.11, *p* =.043), but not with between-strategy variability (*β* = −0.05, *p* =.24). Analyses using EF task scores z-standardized prior to domain- and composite-level aggregation yielded the same pattern of results. Thus, Hypothesis 2 was not supported.

### Results for ER flexibility indices

ER flexibility indices were derived from multilevel models estimating how within- and between-strategy variability covaried with contextual factors (valence, being alone, and being with others). Person-specific slopes from these models were extracted and used as individual-level indices of ER flexibility in subsequent analyses. All models controlled for day since study start, tense arousal, and mean ER strategy endorsement. At the fixed-effects level, within-strategy variability was positively associated with being alone (*β* = 0.09, *p* <.001) and being with others (*β* = 0.10, *p* <.001), whereas valence was not significantly related to within-strategy variability (*β* = −0.002, *p* =.834). Because the context variables were person-mean-centered at the daily level, positive coefficients indicate higher within-strategy variability on days when participants spent more time alone or more time with others than was typical for them (i.e., relative to their own average). Mean ER strategy endorsement showed a strong positive association with within-strategy variability (*β* = 0.87, *p* <.001). For between-strategy variability, none of the contextual predictors showed significant fixed effects (all |β|s < 0.02, all *p*s > 0.05), whereas mean ER strategy endorsement was strongly and positively associated with variability (*β* = 0.90, *p* <.001).

### H3: ER flexibility and unpleasant mood

To test linear and quadratic associations between ER flexibility and unpleasant mood, we regressed mean unpleasant mood on the person-specific flexibility indices derived from the multilevel models. Six flexibility indices were available (three contexts × two variability types). For each variability type (within and between), flexibility in response to valence was tested in separate models (Models 1 and 3), whereas flexibility in social contexts (being alone and being with others) was entered simultaneously (Models 2 and 4). Each model included both linear and quadratic terms (see Table 5).

**Table 5:** Multiple regression results for unpleasant mood (aggregated) being predicted by ER flexibility indices (i.e. individual context–ER variability slopes).

		Unpleasant mood			
		B (SE)	*p*	ß	95% CI ß
Within-strategy Flexibility					
Model 1	(Intercept)	24.19 (1.21)	< 0.001	0.00	[−0.15, 0.15]
	Valence	0.26 (0.25)	0.302	0.10	[−0.09, 0.29]
	**Valence²**	**0.06 (0.03)**	**0.020**	**0.23**	**[0.04**,** 0.42]**
Model 2	(Intercept)	25.43 (1.23)	< 0.001	0.00	[−0.16, 0.16]
	Being alone	0.04 (0.05)	0.458	0.25	[−0.41, 0.90]
	Being alone²	−0.00 (0.00)	0.054	−0.51	[−1.02, 0.01]
	Being with others	−0.04 (0.05)	0.466	−0.23	[−0.87, 0.40]
	**Being with others²**	**0.0004 (0.0002)**	**0.040**	**0.53**	**[0.02**,** 1.03]**
Between-strategy flexibility					
Model 3	(Intercept)	25.59 (1.12)	1.12	0.00	[−0.16, 0.16]
	Valence	0.13 (0.65)	0.65	0.02	[−0.20, 0.24]
	Valence²	0.03 (0.09)	0.09	0.03	[−0.19, 0.26]
Model 4	(Intercept)	25.81 (1.12)	< 0.001	0.00	[−0.16, 0.16]
	Being alone	−0.02 (0.02)	0.378	−0.13	[−0.43, 0.17]
	Being alone²	−0.00 (0.00)	0.928	−0.04	[−0.92, 0.84]
	Being with others	0.00 (0.01)	0.746	0.05	[−0.26, 0.37]
	Being with others²	−0.00 (0.00)	0.995	0.00	[−0.96, 0.95]

Note. Values represent unstandardized regression coefficients (*B*) with standard errors (*SE*), standardized coefficients (β), and 95% confidence intervals (CI). Predictors are person-specific flexibility slopes derived from multilevel models linking contextual factors (valence, being alone, being with others) to within- or between-strategy ER variability, controlling for mean strategy endorsement. Flexibility indices were entered in second-stage regression models separately for valence and social contexts. Significant predictors (*p* <.05) are highlighted in bold.

### ER flexibility based on within-strategy variability

Multiple regression analyses revealed significant nonlinear associations between ER flexibility derived from within-strategy variability and mean unpleasant mood (see Table 5, Models 1–2). For valence-related flexibility, the quadratic term was significant (*B* = 0.06, *SE* = 0.03, *p* =.020; Model 1: *R*^2^ = 0.035), while the linear term was not (*p* =.302). Similarly, flexibility based on being with others showed a significant quadratic association (*B* = 0.0004, *SE* = 0.0002, *p* =.040; Model 2: *R*^2^ = 0.028). Flexibility based on being alone was not significantly associated with unpleasant mood (all *p*s > 0.05).

**Fig. 1:**
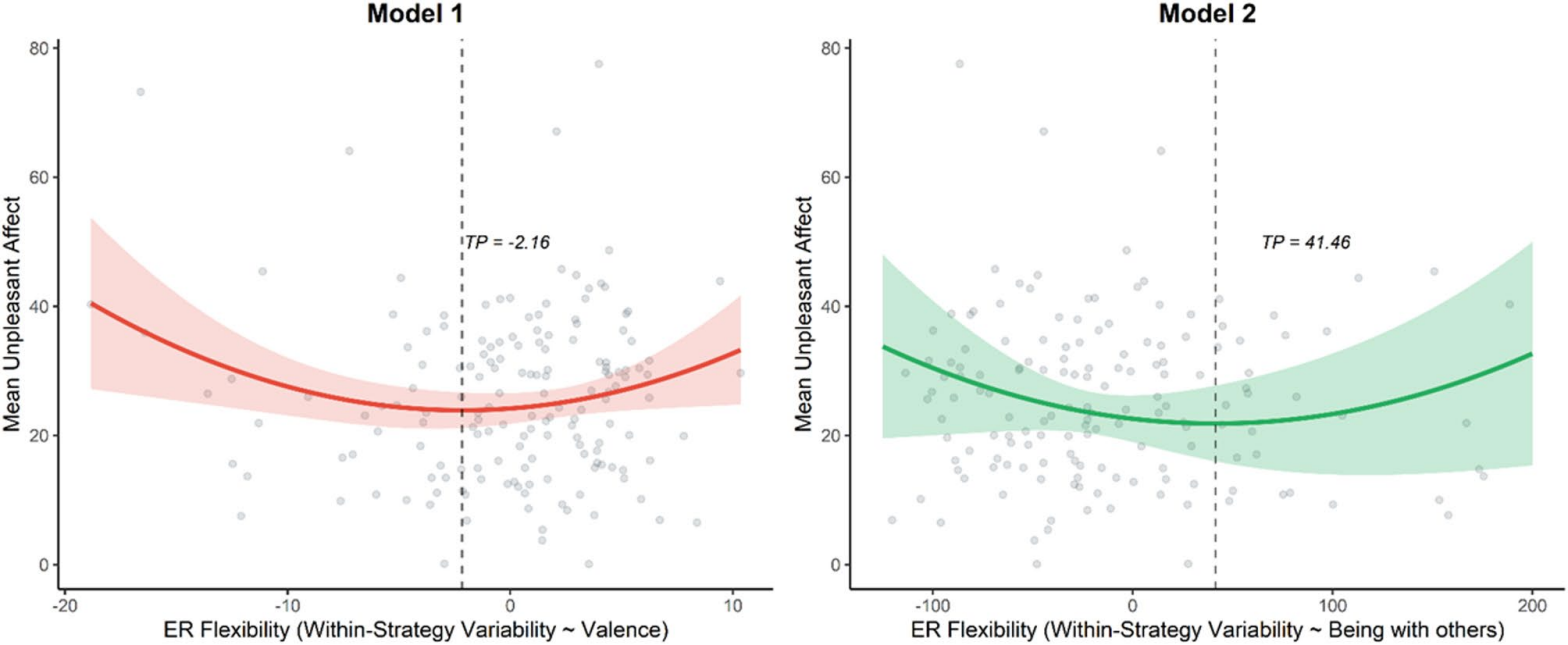
Quadratic associations between within-strategy ER flexibility and unpleasant mood. Note. Panels A and B depict the significant quadratic associations observed in Model 1 (valence) and Model 2 (being with others) between within-strategy ER flexibility and unpleasant mood (aggregated). ER flexibility reflects person-specific slopes obtained from multilevel models estimating the association between within-strategy ER variability and contextual factors (valence, being alone and being with others), while controlling for mean strategy endorsement, arousal, and day of study participation. These slopes were subsequently used as predictors in person-level regression models including linear and quadratic terms. The curves shown in Panels A and B are based on the full regression models reported in the results section; for Model 2, this includes both social-context predictors (being alone and being with others) entered simultaneously. Scatter points represent individual data; curves show the predicted quadratic fits with 95% confidence intervals. Vertical dashed lines indicate the estimated turning points (TP).

Guided by recommendations for interpreting quadratic effects^74^, we interpreted the quadratic terms with regard to their sign and significance, the implied shape across the observed data range, and whether the estimated turning point fell within that range. As illustrated in Fig. 1, the significant positive quadratic terms indicate U-shaped associations, such that unpleasant mood was lowest at moderate levels of ER flexibility. The estimated turning points (TP) were located within the observed range of the respective predictors: TP = −2.16 for valence-related flexibility and TP = 41.46 for flexibility based on being with others.

### ER flexibility based on between-strategy variability

In contrast, ER flexibility indices derived from between-strategy variability were not significantly associated with mean unpleasant mood (see Table 5, Models 3–4). No linear or quadratic terms were significant (all *p*-values> 0.37; Model 3: *R*^2^ < 0.001; Model 4: *R*^2^ = 0.006). Taken together, Hypothesis 3 was partially supported. While the predicted U-shaped relationship was observed for within-strategy flexibility in both valence and shared social contexts (being with others), no such associations were found for the context of being alone or for any indices based on between-strategy variability.

### H4: ER flexibility and depressive symptoms

To test linear and quadratic associations between ER flexibility and depressive symptoms, we regressed PHQ-9 scores on the person-specific flexibility indices derived from the multilevel models. Six flexibility indices were available (three contexts × two variability types). For each variability type (within and between), flexibility in response to valence was tested in separate models (Models 5 and 7), whereas flexibility in social contexts (being alone and being with others) was entered simultaneously (Models 6 and 8). Each model included both linear and quadratic terms (see Table 6).

**Table 6:** Multiple regression results for depressive symptoms being predicted by ER flexibility indices (i.e. Individual Context–ER Variability Slopes).

		Depressive Symptoms			
		B (SE)	*p*	ß	95% CI ß
Within-strategy flexibility					
Model 5	(Intercept)	0.16 (0.31)	0.600	0.00	[−0.16, 0.16]
	Valence	−0.04 (0.06)	0.518	−0.06	[−0.26, 0.13]
	Valence²	−0.01 (0.01)	0.364	−0.09	[−0.29, 0.11]
Model 6	(Intercept)	0.05 (0.32)	0.871	0.00	[−0.16, 0.16]
	Being alone	−0.00 (0.01)	0.946	−0.02	[−0.68, 0.64]
	Being alone²	0.00 (0.00)	0.542	0.16	[−0.36, 0.68]
	Being with others	0.00 (0.01)	0.835	0.07	[−0.57, 0.71]
	Being with others²	−0.00 (0.00)	0.487	−0.18	[−0.69, 0.33]
Between-strategy flexibility					
Model 7	(Intercept)	−0.10 (0.28)	0.721	0.00	[−0.16, 0.16]
	Valence	0.06 (0.16)	0.702	0.04	[−0.18, 0.26]
	Valence²	0.02 (0.02)	0.318	0.11	[−0.11, 0.33]
Model 8	(Intercept)	−0.00 (0.28)	0.990	0.00	[−0.16, 0.16]
	Being alone	−0.00 (0.01)	0.666	−0.07	[−0.37, 0.23] [−0.37, 0.23]
	Being alone²	0.00 (0.00)	0.764	0.13	[−0.74, 1.01]
	Being with others	0.00 (0.00)	0.907	0.02	[−0.30, 0.34]
	Being with others²	−0.00 (0.00)	0.864	−0.08	[−1.04, 0.87]

Note. Values represent unstandardized regression coefficients (*B*) with standard errors (*SE*), standardized coefficients (β), and 95% confidence intervals (CI). Predictors are person-specific flexibility slopes derived from multilevel models linking contextual factors (valence, being alone, being with others) to within- or between-strategy ER variability, controlling for mean strategy endorsement. Flexibility indices were entered in second-stage regression models separately for valence and social contexts.

### ER flexibility based on within-strategy variability

Multiple regression analyses indicated that ER flexibility indices derived from within-strategy variability were not significantly associated with depressive symptoms (see Table 6, Models 5–6). Neither linear nor quadratic terms for flexibility based on valence, being alone, or being with others were significant (all *p*s > 0.36). These within-strategy models explained a negligible proportion of variance in depressive symptoms (Model 5: *R*^2^ = 0.005; Model 6: *R*^2^ = 0.004).

### ER flexibility based on between-strategy variability

Similarly, ER flexibility indices derived from between-strategy variability were not significantly associated with depressive symptoms (see Table 6, Models 7–8). Neither linear nor quadratic terms for flexibility based on valence, being alone, or being with others were significant (all *p*s > 0.31). These models accounted for a negligible proportion of variance in depressive symptoms (Model 7: *R*^2^ = 0.008; Model 8: *R*^2^ = 0.009). Consequently, Hypothesis 4 was not supported, indicating that the U-shaped relationship observed for momentary mood did not extend to depressive symptoms in this sample.

### H5: age, ER variability, and ER flexibility

To examine age-related differences in regulatory dynamics, we conducted a series of linear regression analyses predicting ER variability and flexibility indices from age (results are summarized in Table 7).

### Age and ER variability

When controlling for EF and mean ER strategy endorsement, age was a significant negative predictor of both within-strategy variability and between-strategy variability. Older age was associated with lower within-strategy variability (*b* = − 19.92, *p* <.001) as well as lower between-strategy variability (*b* = − 41.15, *p* <.001). Mean ER strategy endorsement showed a strong positive association with both variability indices (both *p*-values < 0.001), whereas EF was not a significant predictor.

### Age and ER flexibility

Across contexts, age was not consistently associated with ER flexibility, while controlling for EFs. For flexibility based on within-strategy variability, age did not significantly predict flexibility in valence contexts (*b* = − 0.01, *p* =.758), being alone (*b* = − 0.99, *p* =.052), or being with others (*b* = − 0.64, *p* =.184). Similarly, for flexibility based on between-strategy variability, age was not a significant predictor of flexibility in valence contexts (*b* = 0.01, *p* =.275), being alone (*b* = 0.81, *p* =.091), or being with others (*b* = 2.59, *p* =.139). EF were not significantly associated with any flexibility index. Overall, these findings indicate that although older age was robustly associated with lower levels of ER variability, age was not systematically related to ER flexibility across contexts. Any observed associations between age and flexibility were small in magnitude and inconsistent, providing limited evidence for age-related differences in context-dependent ER flexibility. Robustness analyses using ER variability computed without daily aggregation yielded highly consistent results, replicating both the age-related declines and the lack of associations with EF. As models using daily-aggregated indices explained slightly more variance (e.g., *R*^2^ = 0.67 vs. 0.62), suggesting reduced momentary noise, we retained the daily-level approach for all primary analyses. Taken together, Hypothesis 5 was supported. The findings confirm that while older age was robustly associated with lower overall levels of ER variability, context-dependent ER flexibility remained largely stable across the age range studied.

**Table 7:** Age effects on ER variability and ER flexibility.

Variables	Age			
	B (SE)	*p*	ß	95% CI ß
ER Variability				
Within-strategy variability	−19.92 (2.99)	<0.001	−0.35	[− 0.45, − 0.25]
Between-strategy variability	−41.15 (5.36)	<0.001	−0.31	[− 0.38, − 0.23]
ER Flexibility				
Based on within-strategy variability				
Valence	−0.01 (0.03)	0.757	−0.03	[− 0.20, 0.15]
Being alone	−0.99 (0.50)	0.052	−0.17	[− 0.35, 0.00]
Being with others	−0.64 (0.48)	0.184	−0.12	[− 0.29, 0.06]
Based on between-strategy variability				
Valence	0.01 (0.01)	0.275	0.10	[− 0.08, 0.27]
Being alone	0.81 (0.47)	0.091	0.15	[− 0.02, 0.33]
Being with others	2.59 (1.74)	0.139	0.13	[− 0.04, 0.31]

Note. Values represent unstandardized regression coefficients (*B*) with standard errors (*SE*), standardized coefficients (β), and 95% confidence intervals (CI). Age-related differences in ER variability were examined using linear regression analyses separately predicting within- and between-strategy variability from age while controlling for EF and mean ER strategy endorsement. Flexibility predictors represent individual-specific within-person slopes derived from first-stage multilevel models examining the relationship between within- or between-strategy emotion regulation variability and contextual factors (valence, being alone, being with others), while controlling for mean strategy endorsement, arousal and day of study participation. Age-related differences in ER flexibility were examined in separate regression models for each flexibility index, controlling for EF. The influence of mean strategy endorsement was already accounted for during the initial extraction of the flexibility indices.

## Discussion

This preregistered study examined how EF, ER variability, and ER flexibility are related to affective well-being and age in everyday life. Combining standardized online EF tasks with 14 days of ambulatory assessment across a broad age range, we derived person-level indices of ER variability and context-sensitive ER flexibility. Across hypotheses, three main findings emerged. First, EFs showed the expected age-related decline across shifting, inhibition, and updating, yet were largely unrelated to everyday ER variability. Second, ER flexibility demonstrated a differentiated pattern of associations with well-being. Flexibility derived from within-strategy variability showed small but significant quadratic associations with daily unpleasant mood. In contrast, no significant effects were found for between-strategy variability or flexibility related to being alone; furthermore, all flexibility indices remained unrelated to depressive symptoms. Third, age was consistently related to lower ER variability, whereas ER flexibility remained broadly preserved across age. In the following sections, we discuss these findings in the context of cognitive control models of ER, theories of regulatory flexibility, and perspectives on aging and emotional functioning.

### EF and ER variability

Contrary to our preregistered hypotheses, EF performance was not significantly associated with ER variability once age, depressive symptoms, and mean strategy endorsement were controlled for. This adds to a growing body of studies suggesting that associations between EF and ER are selective and contingent on regulatory demands rather than global or uniform^25,27^. At first glance, the absence of robust EF–ER variability associations may appear inconsistent with cognitive control frameworks that conceptualize EFs as core resources supporting variable and flexible ER. However, within the cognitive control framework of ER, EFs are not assumed to directly increase variability or flexibility per se. Instead, they are proposed to support distinct control modes: a shifting mode that enables strategy change and a shielding mode that supports sustained strategy implementation^13,16^. From this perspective, both stable and variable regulation patterns can reflect adaptive control, depending on situational demands and regulatory goals. Accordingly, our findings suggest that higher EF capacity does not necessarily manifest as increased ER variability in daily life. This may be particularly true for shifting; while recent studies suggest its specific relevance for strategy transitions^25,26^, individuals with higher shifting capacity may use this resource to either alternate strategies when demands change or maintain a successful strategy against distractions. When variability is aggregated across days and contexts, the distinct control processes of adaptive switching versus goal-directed persistence may offset one another at the between-person level^75^.

In addition, EFs were assessed as indicators of maximal cognitive performance under standardized and time-limited task conditions with externally defined goals, whereas ER variability reflects typical patterns of self-regulation in emotionally meaningful everyday contexts. Differences between maximal and typical performance are known to attenuate associations between cognitive task measures and real-world behavior, which may further contribute to weak or absent EF–ER variability links in ambulatory designs^76^. This interpretation is consistent with experimental work showing dissociations between EF components and different aspects of regulation performance, as well as trade-offs in shared cognitive resources during regulation^27,30^.

From a cognitive control perspective, these findings suggest that individual differences in global EF capacity may be only weakly related to ER variability in daily life in nonclinical samples. In healthy populations, ER variability may be driven more by situational routines than by executive constraints. In contrast, in clinical samples characterized by pronounced executive deficits, a certain level of EF capacity may support flexible regulation, making EF–ER associations more apparent (cf. ^77^). Our nonclinical findings suggest that EFs were insufficient to account for between-person differences in context-dependent regulation patterns as captured by the present variability indices. Instead, variability may largely reflect stable regulatory habits, preferences, and learned routines that are only indirectly constrained by executive resources. For populations with sufficient cognitive resources, interventions should therefore target specific regulatory skills and monitoring processes to promote adaptive flexibility and ensure a better contextual fit of strategy use, rather than focusing on general cognitive control capacity.

### ER flexibility and affective well-being

The findings reveal a nuanced pattern linking ER flexibility to affective well-being in daily life. Within-strategy flexibility, which reflects moment-to-moment modulation of regulatory intensity, showed significant quadratic associations with unpleasant mood for both situational valence and social context. This pattern aligns closely with theoretical accounts proposing that both insufficient and excessive flexibility may be maladaptive. As suggested by models of regulatory rigidity and emotional instability, very low flexibility may reflect overly stable, context-insensitive responding, whereas very high flexibility may indicate fragmented or weakly goal-guided regulatory adjustment^11–13^. The observed U-shaped associations are therefore consistent with theoretical accounts suggesting that both insufficient and excessive flexibility can be maladaptive, with optimal outcomes emerging at moderate levels of context sensitivity^6,78^.

Although the observed effects were small, they indicate that our operationalization was sufficiently sensitive to capture meaningful individual differences in context-related regulatory patterns, despite the conceptual and methodological challenges inherent in assessing ER flexibility in naturalistic settings^79,80^. This aligns with theoretical accounts positioning flexibility as a regulatory process that supports goal-directed adaptation in specific situations rather than as a global determinant of emotional outcomes^81^ and is consistent with recent ambulatory work showing that flexibility indices are specifically related to momentary affective experience^82^.

At the same time, this pattern was not uniform: flexibility related to being alone, flexibility based on between-strategy variability, and depressive symptoms all showed no corresponding associations, indicating that the observed effects are specific rather than global. One plausible explanation for the absence of effects in between-strategy flexibility is that subtle within-strategy adjustments may capture a more responsive calibration of regulatory effort to momentary demands^83^ than broader categorical shifts. Strategy shifts tend to occur less frequently and are often constrained by situational affordances^78^ or by the high cognitive costs associated with switching between different regulatory goals^17^.

This absence of effects also underscores the importance of contextual granularity. Although situational valence and social context are theoretically meaningful and commonly used in ambulatory research^51^, they may not fully capture the regulatory demands that determine when flexibility becomes adaptive. ER choices are shaped by multiple contextual dimensions, including intensity, controllability, and social-evaluative concerns^1,10^, and social interactions vary widely in their emotional affordances depending on relational closeness, conflict intensity, or perceived threat^84^. Consistent with this, empirical work shows that even a typically adaptive strategy such as reappraisal benefits well-being only when deployed in contexts where it fits situational demands^83,85^. When such finer distinctions are not represented, context-sensitive adaptation may be underestimated, which can attenuate associations with well-being^80^.

Beyond measurement considerations, the differential associations with unpleasant mood and depressive symptoms also indicate important differences between these two indicators of affective functioning. Daily unpleasant mood and depressive symptoms represent conceptually distinct indicators of affective functioning. Daily mood captures short-term fluctuations that closely track situational conditions and momentary regulatory adjustments^70,86^. Depressive symptoms, by contrast, reflect a more enduring affective pattern that unfolds over longer timescales and is shaped by a broader set of contextual, biological, and dispositional influences^71^. Given this, it is reasonable to expect that flexibility indices derived from a two-week ambulatory assessment protocol correspond more strongly to day-to-day variations in affect (see also^87^) than to the broader symptom measure of depressive symptoms. The difference in temporal granularity may explain the absence of associations with depressive symptoms.

The pattern of results also underscores the need to distinguish ER flexibility from overall regulatory intensity. At the descriptive level, ER variability was strongly associated with mean strategy endorsement, whereas contextual features showed comparatively weak or inconsistent effects. Although flexibility indices were computed from person-specific deviations around these means, this pattern suggests that a substantial portion of variability reflects general regulatory engagement rather than targeted contextual adjustment^88^. This is consistent with conceptualizations emphasizing that the adaptiveness of ER depends more on the suitability of the strategy–context match than on variability alone^9,67^.

Flexibility indices are further shaped by several conceptual and methodological decisions. In this study, variability was aggregated across eleven strategies spanning multiple stages of the emotion-generation process^61,89^, capturing a broad regulatory repertoire but potentially obscuring strategy-specific distinctions that may align more directly with individual regulatory goals. Moreover, ER itself serves multiple and sometimes competing goals that extend beyond immediate affect reduction, including instrumental, social, and self-related motives^90^. Depending on which of these goals is prioritized in a given situation, different strategies or intensities may be selected, which complicates efforts to derive a single, context-general index of flexibility. Incorporating momentary assessments of individuals’ regulatory goals may help distinguish when variations in strategy use reflect purposeful, goal-aligned adjustment, thereby sharpening the precision of flexibility indices. Complementary approaches that group strategies into theoretically defined clusters or model person-specific regulatory repertoires may further enhance the extent to which flexibility indices capture the adaptive components of ER^88,91^. Taken together, these considerations suggest that context-sensitive variability—particularly within-strategy adjustments—plays a modest but detectable role in daily affective experience, consistent with perspectives emphasizing balanced rather than maximal flexibility as a hallmark of adaptive emotional functioning^6,78,82,87^.

### Age differences in EF, ER variability, and ER flexibility

As expected, increasing age was associated with lower performance in shifting, inhibition, and updating, although age trends should be interpreted cautiously given the age distribution of the sample (see Supplementary Figure S3). Turning to ER in daily life, higher age was associated with lower levels of both within- and between-strategy ER variability, whereas ER flexibility remained largely stable across a wide age range. The negative association between age and ER variability suggests that older adults show more consistent patterns of ER strategy use in daily life. While reduced variability could, in principle, reflect rigid or inflexible regulation, several aspects of the present findings are more consistent with lifespan perspectives emphasizing adaptive selection and context matching^92,93^. In particular, lower ER variability with increasing age co-occurred with mostly preserved context-dependent modulation of variability and with lower levels of depressive symptoms at the descriptive level (see Table 4), arguing against a generalized loss of regulatory adaptiveness. Lifespan theories propose that older adults increasingly prioritize emotionally meaningful goals and proactively structure their environments to reduce exposure to highly negative or conflictual situations^39,44^. In such relatively stable and self-selected environments, lower ER variability may reflect efficient, well-practiced regulatory routines that are well matched to recurring demands rather than regulatory rigidity. Consistent with this interpretation, daily-life research has reported reduced variability in strategy use at older ages alongside preserved ER effectiveness and well-being^38,42,43^. The present findings provide converging evidence for this account. Although older age was associated with lower overall ER variability, ER flexibility remained relatively stable across the age range studied when accounting for EFs. This aligns with lifespan perspectives suggesting that the ability to context-dependently modulate ER is preserved in older adulthood through processes of selective optimization and compensation^93,94^.

### Limitations and future directions

Several limitations warrant consideration. First, although depressive symptoms were included as a key outcome variable, PHQ scores in the present sample were skewed toward the lower end of the severity range, with relatively few participants reporting moderate to severe symptoms. This restricted range may have limited sensitivity to detect associations between ER flexibility and depressive symptoms, and findings may not generalize to populations with higher symptom burden.

Second, ER strategy use and context variables were assessed via self-report during ambulatory assessment. While this approach provides direct access to subjective regulatory processes in daily life, it may be influenced by self-awareness, reporting biases, and demand characteristics. Future research could complement self-report data with behavioral, physiological, or informant-based measures to triangulate patterns of ER variability and flexibility.

Third, although the sample covered a wide age range, the number of participants above age 60 was comparatively small. Denser sampling in later adulthood is needed to more precisely characterize potential nonlinear age trends in ER variability and flexibility.

Fourth, a methodological issue concerns the high correlation (*r* >.76) between mean ER endorsement and our variability indices. Controlling for mean levels notably strengthened the association between age and variability compared to bivariate correlations (see Tables 4 and Table 7), indicating that age differences in ER variability are partly obscured by interindividual differences in overall strategy intensity. This pattern suggests that variability captures two intertwined components: stable regulatory routines and context-sensitive adjustments. While this control ensures that age effects are not a mere byproduct of strategy frequency, controlling for such a dominant covariate can make the residual variance difficult to interpret. Future research should clarify whether ER variability, once separated from overall frequency, reflects a distinct regulatory capacity or primarily an inherent feature of general regulatory intensity. Similarly, the substantial correlations between our ER flexibility indices and both within- and between-strategy variability (see Tables 4 and Table S1 in the Supplemental material) reflect an inherent conceptual overlap, as flexibility is operationalized as context-dependent adjustment and thus necessarily incorporates variability. However, the high magnitude of these associations indicates that it is empirically challenging to isolate the unique variance of context-sensitive fit from broader patterns of strategy fluctuation. Future research should aim to develop metrics that more clearly differentiate the adaptive quality of these adjustments from the mere frequency or intensity of strategy change.

Fifth, although the key instruments used in the present study are widely applied, the generalizability of these findings across diverse cultural contexts remains to be established. The PHQ-9 has demonstrated stable psychometric performance globally^95^, and the ER flexibility indices employed here are based on core mechanisms of context-sensitive adaptation theorized to be foundational across populations^9^. Similarly, the EF tasks target a cognitive architecture that is considered relatively universal^96^. However, as specific strategy preferences and their social consequences are known to vary across cultures^97^, the normative meaning of certain ER patterns in our sample may not fully generalize^98^.

A final limitation concerns the reliance on aggregated, between-person indicators of ER variability and flexibility. Although such summaries are useful for characterizing stable between-person differences in regulatory functioning, they may obscure the inherently dynamic and context-dependent nature of regulatory processes. Flexibility likely unfolds within individuals across situations and time, whereas person-level summaries necessarily reduce such momentary adaptations to stable aggregate estimates. Relatedly, the EF tasks employed here capture relatively stable, trait-like cognitive capacity under conditions of maximal performance. In combination with known issues of task impurity, this raises the possibility that these measures only partially reflect the momentary cognitive processes engaged during everyday ER. Future research would benefit from integrating intensive longitudinal designs with state-sensitive cognitive assessments to better capture these dynamics.

## Conclusion

This study provides a nuanced picture of how EF, ER variability, ER flexibility, and age relate to everyday affect. As expected, EF declined with age, yet these declines showed little correspondence with either within-strategy or between-strategy ER variability. Age was consistently associated with lower ER variability, whereas flexibility remained broadly stable across the age range studied. ER flexibility was not uniformly linked to well-being. Only within-strategy flexibility showed quadratic associations with daily unpleasant mood, whereas flexibility related to being alone and between-strategy flexibility showed no corresponding effects, and no associations were found for depressive symptoms. This suggests that moment-to-moment adjustments in regulatory intensity—not broader shifts across strategies—are most relevant for daily affective experience. Together, these findings suggest that adaptive ER cannot be reduced to “more executive control” or “more flexibility.” Instead, the adaptiveness of ER appears to depend on how well regulatory responses match situational demands. Future research would benefit from refining the operationalization of such strategy–context matches and incorporating finer-grained contextual and goal-related assessments to more precisely capture adaptive regulatory dynamics.

## Supplementary Information

Below is the link to the electronic supplementary material.

Supplementary Material 1

## Data Availability

The anonymized datasets generated and analyzed during the current study are available in the OSF repository: https://osf.io/s2hfg/. The repository contains anonymized prompt-level ambulatory assessment data, anonymized person-level questionnaire data, anonymized executive-function task scores, and a data dictionary.
