# Supplementary material for: Emotion regulation variability and flexibility in daily life show distinct associations with well-being, age, and executive functions
Source: Sci Rep. 2026 Jun 15;16:18531. doi: 10.1038/s41598-026-57813-7 (PMC13269914; doi:10.1038/s41598-026-57813-7)
Supplement: Supplementary file 1 — Supplementary Material 1 [file 41598_2026_57813_MOESM1_ESM.docx]

**Supplementary Information
*Emotion regulation variability and flexibility in daily life show distinct associations with well-being, age, and executive functions***

## Figure S1

##
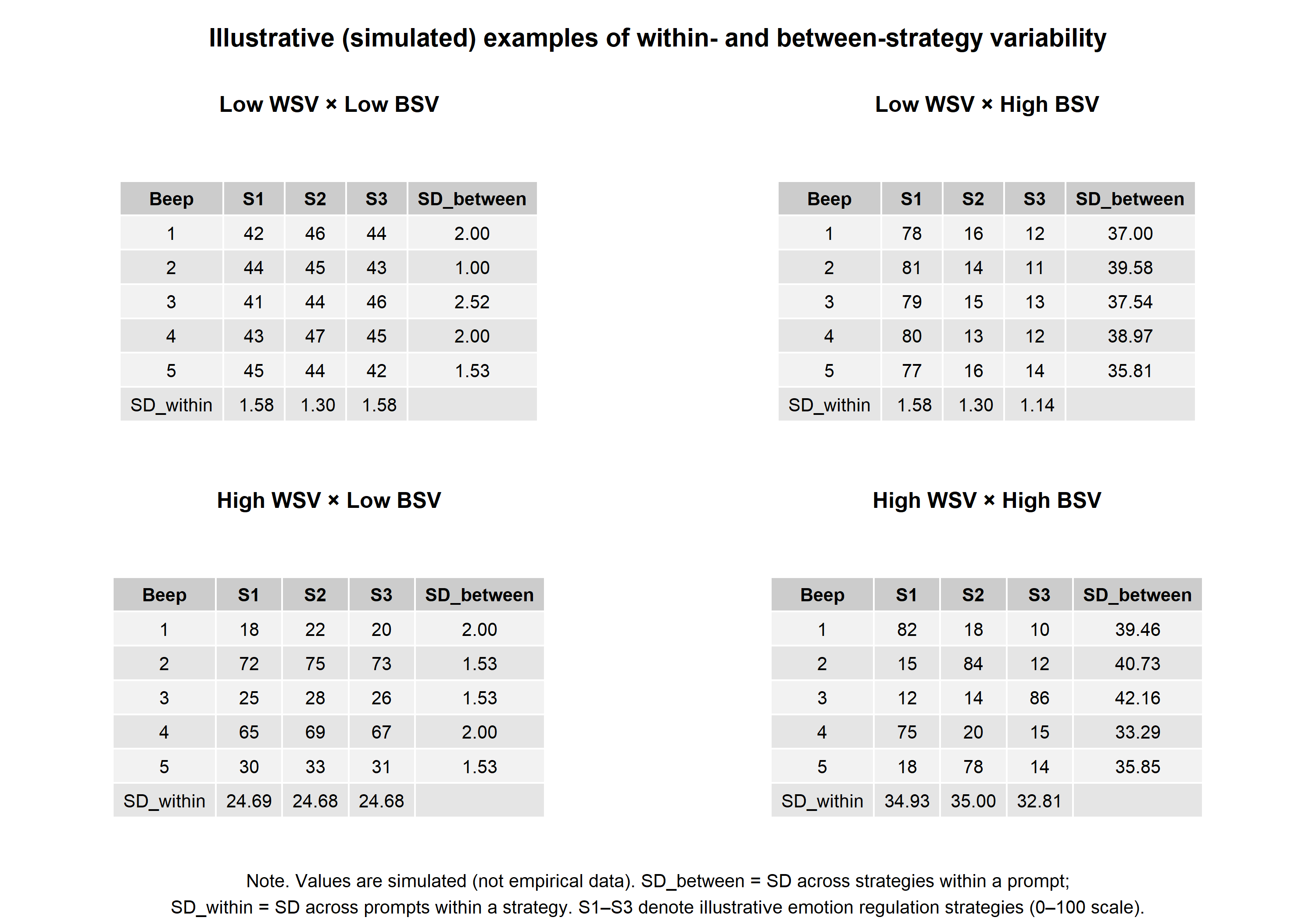
*Illustrative examples of within- and between-strategy variability*

## Figure S2 *Distribution of Depressive Symptom Severity*


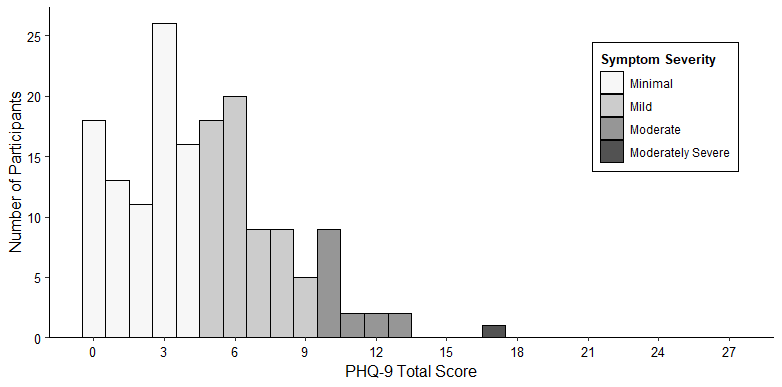


*Note. N*=161. PHQ-9 total scores showed a characteristic right-skewed distribution. Frequencies (n) and percentages per category were: Minimal (0–4; n=84, 52.2%), Mild (5–9; n=61, 37.9%), Moderate (10–14; n=15, 9.3%), and Moderately Severe (15–19; n=1, 0.6%).

## Figure S3 *Executive Function Performance as a Function of Age*


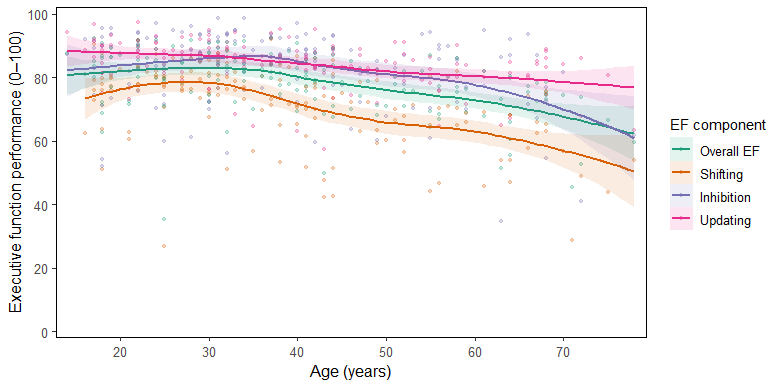


**Note.** Executive function performance (overall EF, shifting, inhibition, updating) as a function of age. Lines show LOESS smoothers with 95% confidence intervals.

## Table S1

## *Extended Correlation Matrix of Study Variables*

| Variable | 1 | 2 | 3 | 4 | 5 | 6 | 7 | 8 | 9 | 10 | 11 | 12 | 13 | 14 | 15 | 16 | |
| --- | --- | --- | --- | --- | --- | --- | --- | --- | --- | --- | --- | --- | --- | --- | --- | --- | --- |
| 1. Age | — |  |  |  |  |  |  |  |  |  |  |  |  |  |  |  | |
| 2. PHQ-9 | -.35* | — |  |  |  |  |  |  |  |  |  |  |  |  |  |  | |
| 3. EF overall | -.47* | .04 | — |  |  |  |  |  |  |  |  |  |  |  |  |  | |
| 4. Shifting | -.50* | .08 | .85* | — |  |  |  |  |  |  |  |  |  |  |  |  | |
| 5. Inhibition | -.24* | -.01 | .77* | .43* | — |  |  |  |  |  |  |  |  |  |  |  | |
| 6. Updating | -.41* | .02 | .78* | .63* | .41* | — |  |  |  |  |  |  |  |  |  |  | |
| 7. Within-strategy variability | -.06 | .06 | -.06 | -.10 | -.12 | -.00 | — |  |  |  |  |  |  |  |  |  | |
| 8. Between-strategy variability | .06 | .02 | -.12 | -.19* | -.14 | -.02 | .91* | — |  |  |  |  |  |  |  |  | |
| 9. Mean ER strategy endorsement | .25* | -.05 | -.21* | -.28* | -.16* | -.09 | .76* | .91* | — |  |  |  |  |  |  |  | |
| 10. Unpleasant mood | -.11 | .44* | -.09 | -.05 | -.10 | -.06 | .07 | .00 | -.08 | — |  |  |  |  |  |  | |
| 11. Flexibility (within; valence) | .02 | -.03 | -.07 | .02 | -.08 | -.05 | -.47* | -.43* | -.34* | .07 | — |  |  |  |  |  | |
| 12. Flexibility (within; alone) | -.07 | .06 | -.04 | -.10 | -.07 | .03 | .95* | .93* | .80* | .04 | -.54* | — |  |  |  |  | |
| 13. Flexibility (within; with others) | -.07 | .07 | -.01 | -.08 | -.03 | .03 | .87* | .84* | .70* | -.00 | -.78* | .94* | — |  |  |  | |
| 14. Flexibility (between; valence) | -.06 | -.02 | .12 | .18 | .12 | .01 | -.90* | -.99* | -.91* | .02 | .44* | -.94* | -.85* | — |  |  | |
| 15. Flexibility (between; alone) | .09 | -0.08 | -.00 | -.03 | .01 | -.06 | -.27* | -.25* | -.23* | -.06 | -.06 | -.22* | -.14 | .27* | — |  | |
| 16. Flexibility (between; with others) | -.00 | -0.07 | .06 | .09 | .09 | -.05 | -.82* | -.87* | -.80* | .00 | .31* | -.81* | -.71* | .88* | .65* | — | |
| *Note***.** *N* = 161. Spearman rank-order correlations are reported at the between-person level. AA-based variables reflect averages across the two-week assessment period; EF and depressive symptoms were assessed once. PHQ-9 = Patient Health Questionnaire–9, assessing depressive symptoms. * p < .05. *p*-values were adjusted using the Benjamini–Hochberg false discovery rate procedure. | | | | | | | | | | | | | | | | |  |
